# Supplementary material for: A mobile-optimized artificial intelligence system for gestational age and fetal malpresentation assessment
Source: Commun Med (Lond). 2022 Oct 11;2:128. doi: 10.1038/s43856-022-00194-5 (PMC9553916; doi:10.1038/s43856-022-00194-5)
Supplement: Supplementary file 1 — Description of Additional Supplementary Files [file 43856_2022_194_MOESM1_ESM.pdf]

Description of Additional Supplementary Files

File Name: Supplementary Data 1

Description: Source data for figures 2 and 3
