# Supplementary material for: A mobile-optimized artificial intelligence system for gestational age and fetal malpresentation assessment
Source: Commun Med (Lond). 2022 Oct 11;2:128. doi: 10.1038/s43856-022-00194-5 (PMC9553916; doi:10.1038/s43856-022-00194-5)
Supplement: Supplementary file 3 — Supplementary Material [file 43856_2022_194_MOESM3_ESM.pdf]

Supplementary Methods

Model Architecture

Our deep learning models consist of convolutional and recurrent components (Supplementary Data Figure 3). The convolutional component uses the MobileNetV2 network[1] as a feature extractor for each video frame. This network was selected from a set of alternatives, including MobileNetV3[2] and Resnet[2], based on evaluations using the tuning data set. In our experiments, MobileNetV2 provided the best tradeoff between accuracy and runtime performance. The final feature layer of MobileNetV2 generates a sequence of image embeddings which are then processed by a grouped convolutional LSTM cell[3]. Since the recurrent connections in the model only operate on condensed features extracted from individual frames by MobileNetV2 and don't require the original frames, the forward pass requires relatively little memory, helping to enable deployment on mobile devices. LSTM state and output spatial feature maps are spatially average-pooled and transformed via a fully connected layer before final sigmoid, linear, or softplus units (depending on the prediction task) to compute the model's predictions. The model's final prediction is the output of these units after processing the last frame in the video sequence.

We specialized this architecture for two distinct diagnostic models: gestational age prediction and fetal malpresentation screening. We defined gestational age prediction as a regression problem in which the model produces an estimate of gestational age, measured in days, for each video sequence. The model operates on log-transformed labels and uses linear output units. To make predictions, we exponentiate the raw model output to recover the original scale. The gestational age model also provides an estimate of expected variance for each video sequence, which is generated by an additional softplus model output. This expected variance is inverted to give the confidence feedback score discussed in the main text. We use the mean-variance regression loss function proposed in [4,5] to jointly train the model's log-transformed age and expected variance outputs. The combined loss function has the following form:

$$\sum_i \frac{(f(x_i) - y)^2}{g(x_i)} + \log g(x_i)$$

where $f(x_i)$ is the gestational "log-age" prediction for video clip i, y is the (log-transformed) age label, and $g(x_i)$ is the model's estimate of expected variance for the clip.

The fetal malpresentation model predicts a binary value indicating whether the fetus is in cephalic (head down) versus non-cephalic presentation. This output is produced by a sigmoid unit and the model is trained with a standard binary cross-entropy classification loss function.

Data Preprocessing

Ultrasound videos have variable physical scale-- the anatomical size associated with image pixels varies according to depth (frequency) settings of the ultrasound hardware device. The physical scale value, typically in units of centimeters per pixel, is calculated by the ultrasound device and provided as metadata along with recorded video. Hardware depth settings are fixed

during blind sweeps and therefore the physical scale value is constant throughout the duration of each video clip, but may vary substantially across patient studies. We denote the video's physical scale as α_i below.

We found that model performance improves dramatically in the tune set when video frames are rescaled to a constant scale value α across the training set. The rescaling procedure first uses bilinear interpolation to resize the number of pixels in each frame according to α_i/α, which normalizes the per-pixel physical scale. Then the resized image is cropped or padded to fixed height and width. The scale constant α, height, and width were chosen by visually inspecting training set images to ensure that relevant ultrasound image details were not excessively cropped, but otherwise these values were not extensively tuned. The same image rescaling procedure is performed as a pre-processing step during model inference. The gestational age model benefits from high resolution images, and we used 576 x 432 pixels with α=0.0333 centimeters/pixel. The fetal malpresentation model operates with lower resolution, and we used 320 x 240 pixels with α=0.06 centimeters/pixel. The lower resolution setting for the fetal malpresentation model is a technical tradeoff to accommodate training on longer video sequences (described below).

Blind sweep video sequences are divided into multiple equal length clips that correspond to a fixed LSTM sequence length, and each clip serves as an independent training example. Video sequences are padded with zero valued image frames when necessary to arrive at an integral number of fixed length clips. We also apply temporal subsampling in order to reduce computational load during inference and to ensure that clips capture enough context across the span of the sweep motion. For the gestational age model, we use ½ temporal subsampling and clips of 24 frames. We find that aggregating predictions from multiple short clips works well for gestational age estimation (see Mobile Inference section below for details on our aggregation method). The fetal malpresentation model benefits from longer frame sequences that capture the full duration of the blind sweep video. We use ⅓ temporal subsampling and 100 frame clips in order to provide the model with enough spatial context regarding the presentation of the fetus.

The two models required different data preprocessing due to differences in the nature of the estimation problems. Gestational age can be reasonably estimated from short video clips and these estimates can be aggregated together to improve performance. Fetal malpresentation can not be determined by looking at a short sequence or individual images. Judging the spatial orientation of the fetus requires processing complete blind sweep videos to assess spatial relationships between anatomical regions.

Training

The gestational age regression model uses the gestational age ground truth associated with the case as the training label for all video clips within the case. We use both blind sweeps and biometry fly-to videos from the FAMLI dataset during gestational age training. The fetal malpresentation model uses only second and third trimester cases, since fetal presentation is not well defined during early pregnancy. We restrict training to blind sweep videos only. For each training set case, fetal presentation is specified as one of four possible values by an expert

sonographer (cephalic, breech, transverse, oblique.) We transform this to a binary training label by grouping breech, transverse, and oblique presentations into a single non-cephalic category.

The MobileNetV2 feature extractor's weights were pre-trained on ImageNet[6] data, and further refined along with the rest of the model weights during training on ultrasound data. Training was done using TensorFlow running on third generation tensor processing units with a 4x4 topology. During training we applied random data augmentation: horizontal and vertical flips as well as random image crops were uniformly applied across all frames within the training video clip. The LSTM state and output tensors have channel width equal to 512. We applied dropout with a keep probability of 0.863 for the gestational age model and 0.8 for the fetal malpresentation model. Model parameters were learned via AdamW optimization [7] with a batch size of 8. Learning rates were decreased from an initial starting point according to a linear ramp based on the training step number. The gestational age model began with a learning rate of 4.58e-4 and ended with 4.58e-7 after 1 million training steps. The fetal presentation model began with learning rate 3.14e-5 and ended with 3.14e-7 after 300,000 training steps. Dropout probabilities and learning rate schedules were selected based on performance evaluations on the tuning set, using the Vizier optimization system[8].

Mobile Device Inference

Our trained models were converted to a suitable format for deployment on mobile devices using the TensorFlow Lite (TF-Lite) framework. After training in TensorFlow, we replaced the input layer in the model graph with a TF-Lite compatible input layer. The TF-Lite model performs inference on a single image at each time step, and accepts the current image and previous LSTM state from the previous time step as inputs.

During inference we used the same image re-scaling and temporal subsampling settings that are used in training, but we omitted random data augmentation. All reported model evaluation results were produced by running inference with the converted model in a TF-Lite runtime, which provides identical results independent of the underlying hardware used for evaluation.

Each case contains multiple blind sweep videos of varying length. We divided them into equal length video clips that match the LSTM sequence length used during training. We used the model's prediction on the final frame of the sequence as the single prediction for the clip. Predictions for all video clips in each case were aggregated together to generate a single prediction for the case. The gestational age model uses an inverse variance weighting procedure [9] to combine the clip-level predictions x_c, into a case-level mean gestational age $\overline{x}$ using estimated variance σ_c of each clip

$$\bar{x} = \frac{\sum_c x_c/\sigma_c^2}{\sum_c 1/\sigma_c^2}$$

Since the model fits log-transformed gestational age $f = log(GA)$ and the variances *g* are fitted to a normal distribution of *f,* the true variance of gestational age σ_c needs correction using the log-normal distribution variance formula

$$\sigma_c = [\exp(g^2) - 1]\exp(2\mu + g^2)$$

where the mean $\mu = f_c$. This aggregation method was used to accommodate studies that contain either 6 or 2 sweeps (see "Simplified sweep evaluation" section, in the main article). When the protocol consists of fewer sweeps, there are fewer equal length video sequence predictions available for aggregation but the operation of the underlying model is the same.

The fetal presentation model generates one prediction per blind sweep video, and we average the model's sigmoid probability outputs to generate a case-level probability that the fetus is in non-cephalic presentation.

We selected our model architecture to optimize for runtime performance on standard mobile devices, such that the model can perform real-time inference on a live ultrasound video stream. We explored model weight quantization and inference delegate configurations, but settled on non-quantized floating point model weights since performance was sufficient when using modern mobile phones with graphics processing units or neural network acceleration libraries. Runtime performance was evaluated on the following smartphone devices: Google Pixel 3 which uses the Qualcomm SnapDragon 845 chipset (Kryo 385 CPU, Adreno 630 GPU), and Google Pixel 4, Samsung Galaxy S10, and Xiaomi Mi 9 phones which use the Qualcomm SnapDragon 855 chipset (Kryo 485 CPU, Adreno 640 GPU). Benchmarking results are provided in Table 3, in the main article.

Supplementary Fig. 1: STARD Diagrams. a: Blind sweeps performed by trained sonographers (FAMLI study). **b:** Blind sweeps performed by midwives (novice ultrasound operators.)

a

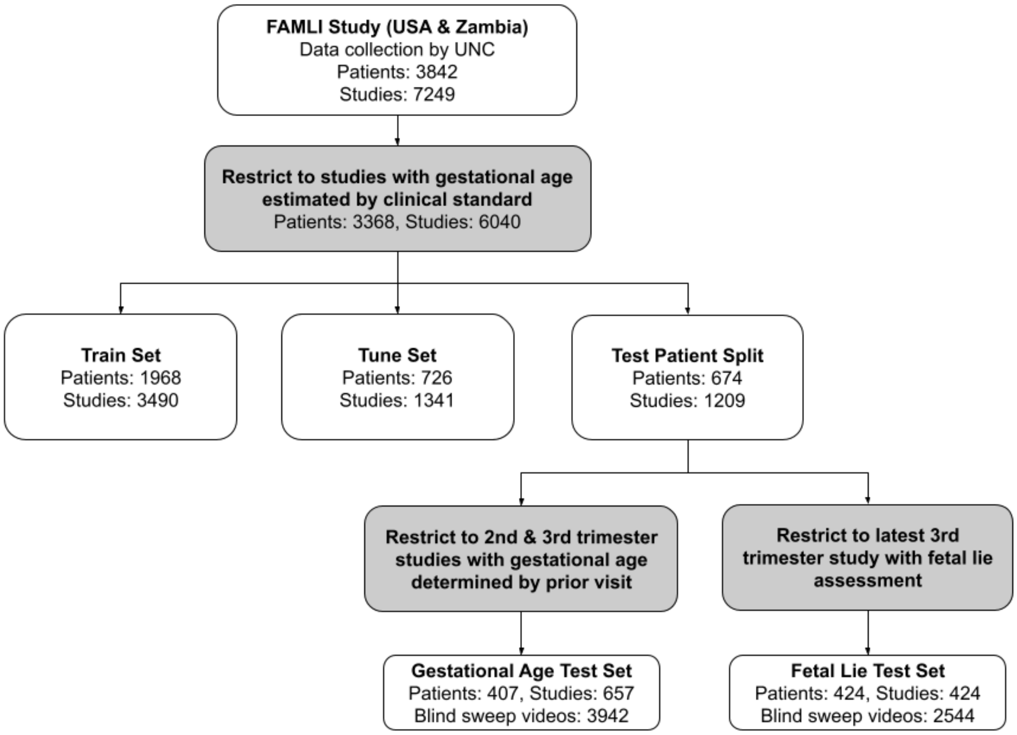

b

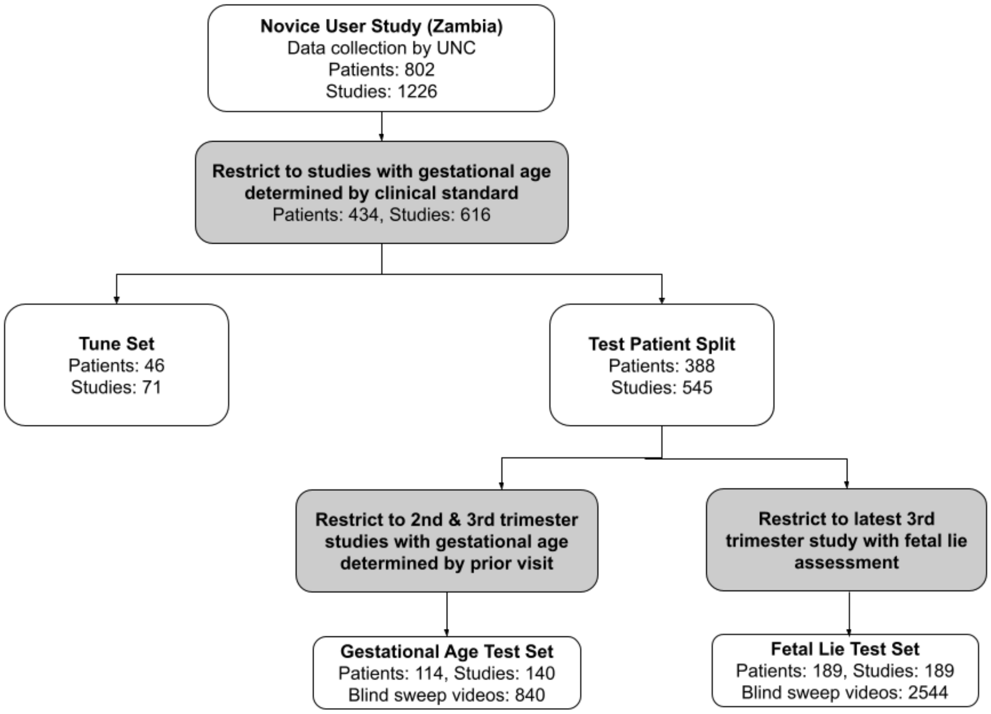

Supplementary Fig. 2: Blind sweep and Biometry Images. a, left: Abdominal circumference measured by a sonographer. The clinical standard for estimating gestational age requires measurements of fetal anatomy captured from precise standardized viewpoints. **a, center & right:** Blind sweep example video frames. Blind sweep AI models do not require standardized anatomical views and videos may be acquired by ultrasound operators with little training. **b, left:** Example video frame acquired by a high end ultrasound device (GE Voluson family). See also row A for examples from SonoSite M-Turbo. **b, center, right:** Example video frames acquired by a low cost portable ultrasound device (Butterfly IQ). **c, left & center left:** Examples of blind sweep video frames with high feedback score (randomly selected from the top score percentile) for the gestational age model. The fetal abdomen (left) and head (center left) are visible. **c, center right & right:** Examples of blind sweep video frames with low feedback score (randomly selected from the lowest score percentile) for the gestational age model. The fetus is not visible in either example.

a

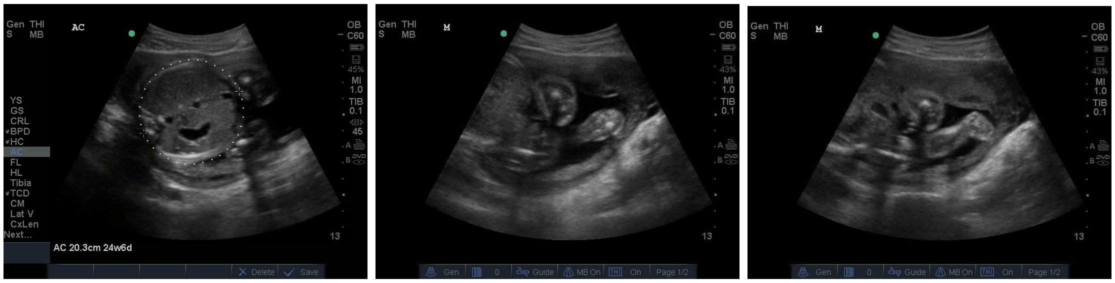

b

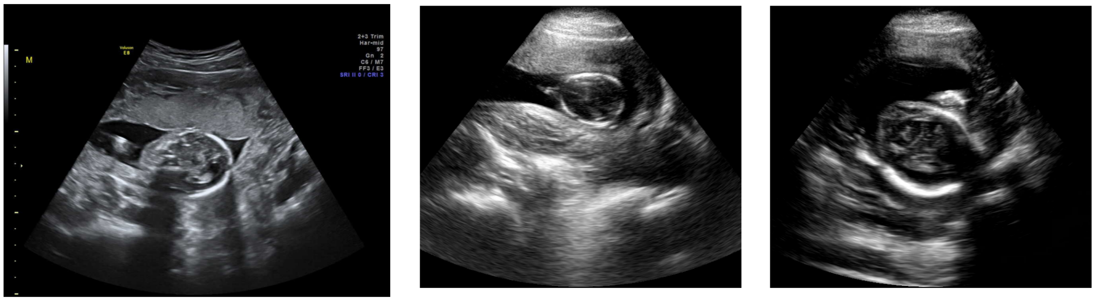

c

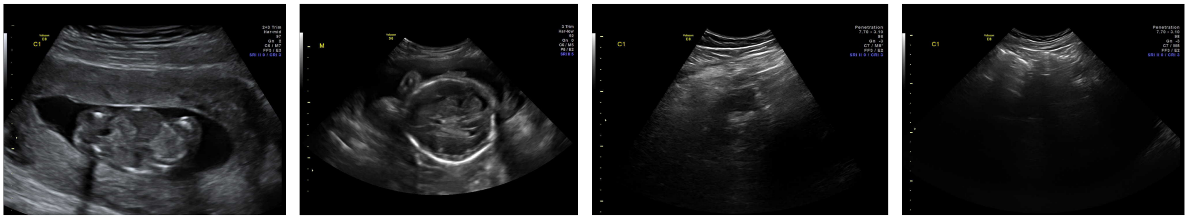

Supplementary Fig. 3: Artificial Intelligence Model Architecture. The MobileNetV2 network [1] is used as a feature extractor applied to each video frame. The final feature layer of MobileNetV2 generates a sequence of image embeddings which is then processed by the grouped convolutional LSTM cell[3]. This architecture was selected for its suitability for deployment on mobile phones.

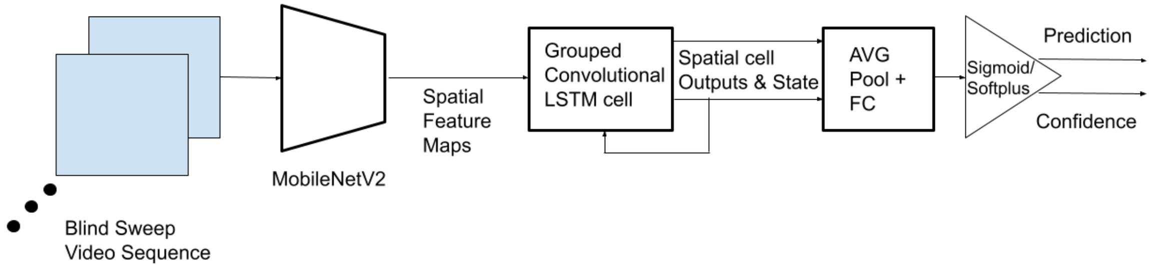

Supplementary Table 1: Characteristics of study participants.

Variable	Sonographer group (n=407)	Novice group (n=114)
Participant age at enrollment (± standard deviation)	28.8 ± 5.6 years	28.0 ± 5.8 years
Gestational age at first study visit (± standard deviation)	159.4 ± 49.7 days	167.8 ± 47.4 days
Total number of study visits during second trimester (%)	273 (41.6%)	40 (28.8%)
Total number of study visits during third trimester	384 (58.5%)	99 (71.2%)
Fetal malpresentation (%)	10.3%	11.1%

Supplementary Table 2: Average Length of Blind Sweeps Performed by Novices. See Figure 1B for a depiction of the blind sweep types.

Blind sweep type	Mean Length ± standard deviation
M	10.0 ± 4.2 seconds
R	10.1 ± 4.1 seconds
L	10.2 ± 4.2 seconds
C1	9.6 ± 3.8 seconds
C2	9.7 ± 4.6 seconds
C3	9.3 ± 3.5 seconds

Supplementary References

1. Sandler, M., Howard, A., Zhu, M., Zhmoginov, A. & Chen, L.-C. MobileNetV2: Inverted Residuals and Linear Bottlenecks. *2018 IEEE/CVF Conference on Computer Vision and Pattern Recognition* (2018) doi:10.1109/cvpr.2018.00474.
2. Howard, A. *et al.* Searching for MobileNetV3. *2019 IEEE/CVF International Conference on Computer Vision (ICCV)* (2019) doi:10.1109/iccv.2019.00140.
3. Liu, M., Zhu, M., White, M., Li, Y. & Kalenichenko, D. Looking Fast and Slow: Memory-Guided Mobile Video Object Detection. *arXiv [cs.CV]* (2019).
4. Nix, D. A. & Weigend, A. S. Estimating the mean and variance of the target probability distribution. *Proceedings of 1994 IEEE International Conference on Neural Networks (ICNN'94)* (1994) doi:10.1109/icnn.1994.374138.
5. Lakshminarayanan, B., Pritzel, A. & Blundell, C. Simple and Scalable Predictive Uncertainty Estimation using Deep Ensembles. *arXiv [stat.ML]* (2017).
6. Deng, J. *et al.* ImageNet: A large-scale hierarchical image database. in *2009 IEEE Conference on Computer Vision and Pattern Recognition* (IEEE, 2009). doi:10.1109/cvpr.2009.5206848.
7. Loshchilov, I. & Hutter, F. Decoupled Weight Decay Regularization. *arXiv [cs.LG]* (2017).
8. Golovin, D. *et al.* Google Vizier. *Proceedings of the 23rd ACM SIGKDD International Conference on Knowledge Discovery and Data Mining* (2017) doi:10.1145/3097983.3098043.
9. Cochran, W. G. The Combination of Estimates from Different Experiments. *Biometrics* **10**, 101–129 (1954).
